# Assessing association of dental caries with child oral impact on daily performance; a cross-sectional study of adolescents in Copperbelt province, Zambia

**DOI:** 10.1186/s12955-023-02127-9

**Published:** 2023-05-18

**Authors:** Severine N Anthony, Febronia K Kahabuka, Nancy Birungi, Anne N Åstrøm, Seter Siziya, Hawa S Mbawalla

**Affiliations:** 1 Department of Dental Clinical Sciences, Michael Chilufya Sata School of Medicine, The Copperbelt University, Ndola, Zambia; 2 Department of Orthodontics, Paedodontics and Community Dentistry, School of Dentistry, Muhimbili University of Health and Allied Sciences, Dar es Salaam, Tanzania; 3 Department of Clinical Dentistry, Faculty of Medicine, University of Bergen, Bergen, Norway; 4 Oral Health Services of Western Norway, Bergen, Norway; 5 Department of Public Health, Michael Chilufya Sata School of Medicine, The Copperbelt University, Ndola, Zambia

**Keywords:** C-OIDP, Dental caries, DAG, Adolescents

## Abstract

**Background:**

Dental caries and child oral impact on daily performance (C-OIDP) have been linked in several studies. However, the studies used caries indices, which limit the ability to examine how C-OIDP prevalence varies across various stages of the dental caries process. Furthermore, cross-cultural differences between Zambia and other African countries where the C-OIDP instrument has been widely used necessitate testing its pychometric properties. This study’s primary aim was to evaluate the association between dental caries and C-OIDP. Secondarily, the study reports the psychometric properties of the C-OIDP index among Zambian adolescents.

**Methods:**

A cross-sectional study was conducted between February and June 2021 among grade 8–9 adolescents in Copperbelt province, Zambia. A multistage cluster sampling method was used to select participants. Using a pretested self-administered questionnaire, socio-demographics, oral health behaviors, self-reported oral health, and C-OIDP were evaluated. The test-retest and internal consistency reliability of the C-OIDP were evaluated. The Caries Assessment and Treatment Spectrum (CAST) was used to evaluate dental caries. Adjusted odd ratios and 95% confidence intervals were used to evaluate the association between dental caries and C-OIDP after adjusting for confounders identified by a directed acyclic graph.

**Results:**

Among 1,794 participants, 54.0% were females, while 56.0% were aged 11–14 years. About a quarter (24.6%) had one or more teeth at the pre-morbidity stage, 15.2% at the morbidity, 6.4% at the severe morbidity and 2.7 at the mortality stage. The internal consistency reliability of the C-OIDP Cohen’s Kappa was 0.940, while the Kappa coefficients of the C-OIDP items ranged from 0.960 to 1.00. Participants with severe caries had a high prevalence of C-OIDP, with rates for morbidity, severe morbidity, and mortality stages being 49.3%, 65.3%, and 49.3%, respectively. Oral impacts were 2.6 times (AOR 2.6, 95% CI 2.1–3.4) more likely to be reported by participants with dental caries than those without caries.

**Conclusions:**

Dental caries was associated with high reporting of C-OIDP, and C-OIDP prevalence was high among participants in the severe stages of the caries process. The English version of the C-OIDP demonstrated adequate psychometric characteristics for assessing OHRQoL among Zambian adolescents.

**Supplementary Information:**

The online version contains supplementary material available at 10.1186/s12955-023-02127-9.

## Background

The shift in perception of oral health towards a state of complete physical, mental, and social well-being and not merely the absence of diseases has led to studies assessing oral health-related quality of life (OHRQoL). Oral Impact on daily performance (OIDP) is the most common OHRQoL measurement tool for assessing the extent to which an individual’s daily activities may be affected by oral problems [1, 2]. A child version of the OIDP inventory, (C-OIDP), has been developed after modifications of the adult version, to fit children’s cognitive level of development [3]. The C-OIDP assesses eight oral impacts, of which difficulties with eating and socializing are respectively the most and least frequently reported impacts [2]. Despite widespread usage of the C-OIDP instrument and evidence of excellent psychometric qualities in diverse cultural contexts in Sub-Saharan Africa (SSA) [4], cross-cultural differences necessitate testing it before use in regions like Zambia, where it is employed for the first time. Furthermore, no study has investigated the association between dental caries and C-OIDP among school-going children in Zambia. The only study done in Zambia investigated the impact of malocclusions on OHRQoL using the Child Oral Health Impact Profile short-form instrument (COHIP-SF19). However, the impacts of other prevalent oral diseases were not investigated [5]. Another small-scale cross-sectional study [6] used the adult version of the OIDP questionnaire to examine the effects of oral diseases among patients visiting a dental clinic in Livingstone, Zambia.

Although the association between dental caries and C-OIDP has been well established in previous research [4, 7], the current study is intended to contribute to understanding how the prevalence of C-OIDP varies across different levels of the CAST index, which reflect caries severity. Adolescents with dental caries report oral impacts one to five times more frequently than those without dental caries, according to prior studies [4]. Independent of clinical measures of oral diseases such as dental caries and gingivitis, C-OIDP has been associated with sociodemographic, behavioral, and psychological factors in various populations, showing that the poor, the socially disadvantaged, and those living in rural areas are most negatively affected [2, 7, 10]. Females are also reported to be more affected than males [2] although some studies did not find any sex differences [4, 8, 9]. Oral impacts are reported more frequently by older adolescents than by younger ones, according to some studies in SSA [8, 10], although some studies report no age difference in the occurrence of oral impacts [11, 12]. Studies from high-income countries have also found a similar age distribution of C-OIDP [2].

This study evaluated the association between dental caries, and child oral impact on daily performance (C-OIDP) among adolescents enrolled in Zambian public secondary schools. The study also reports the psychometric properties of the C-OIDP index among adolescents in Zambia.

## Methods

### Study design, population, and setting

**Fig. 1:**
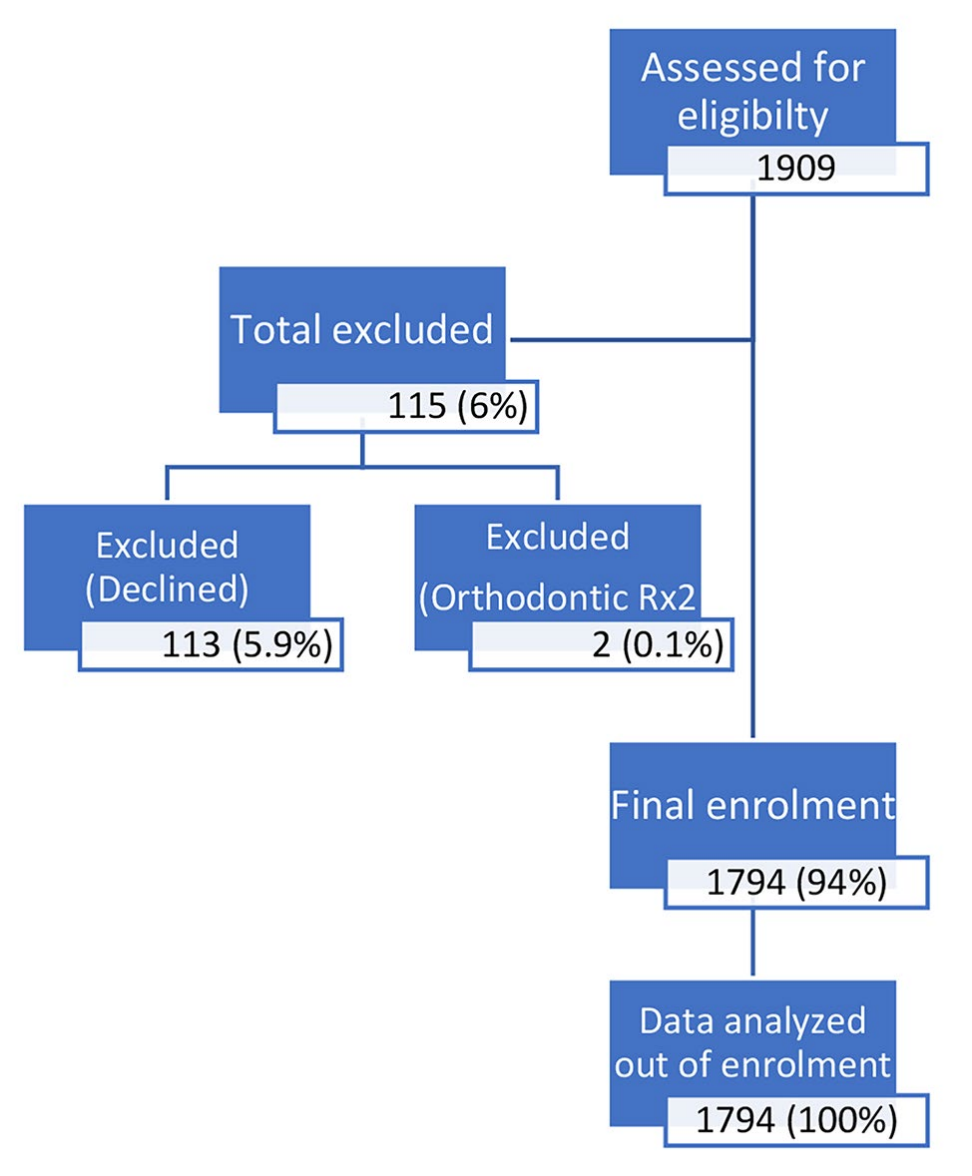
Participants’ enrollment chart

A cross-sectional study was conducted in February-June 2021 at 22 public secondary schools in the Copperbelt Province of Zambia. A total of 1,909 adolescents were assessed for eligibility, 115 (6.0%) were excluded due to being under fixed orthodontic treatment or lack of consent, resulting in the enrollment of 1,794 participants (Fig. 1). The number of participants per school (cluster units) ranged from 43 to 144 (Appendix 2).

### Sample size and sampling procedure

The study reports baseline data from a cluster randomized controlled trial where the sample was estimated to be 1,760 participants, assuming: a 95% confidence level, 85% power, 5% margin of error, 20% expected mean change in dental caries, 0.001 inter-cluster correlation, a cluster size of 80 and a mean DMFT of 1.34 found in a previous study in Zambia [13]. The Copperbelt Province was conveniently selected out of the ten provinces of Zambia at the first stage. The choice of Copperbelt Province was based on fitting the study into existing community dentistry training at the university where the principal investigator is based. The second stage involved proportionate stratified random sampling of three districts out of ten districts in Copperbelt Province; seven being rural and three urban, where Ndola is an urban district while Masaiti and Mpongwe are rural districts. In the third stage, a total of 22 out of 35 public secondary schools were proportionately randomly selected based on the number of schools in each district (21:35, = 13 schools for Ndola district; 8:35, = 5 schools for Masaiti district; and 6:35, = 4 schools for Mpongwe district). In the fourth stage, all adolescents in grades 8 and 9 attending the selected schools were invited to participate in the study.

### Survey Instruments and measurements

#### Questionnaire

A pretested, self-administered paper-based questionnaire constructed in English was used to collect information regarding the C-OIDP and covariates (sociodemographic and oral health behaviors). The questionnaire was pre-tested at one secondary school in Ndola, which was not part of the study, by administering it to 50 adolescents.

### Child-oral impact on daily performance (C-OIDP)

#### Psychometric properties of C-OIDP

The participants in the pilot test were requested to provide feedback on the items’ clarity, difficulty, and arrangement as part of the assessment of the face and content validity of the English version of the C-OIDP questionnaire. Construct validity was tested by evaluating the association between other variables that are linked to overall C-OIDP and C-OIDP items, such as self-rated oral health and clinically examined dental caries. The internal consistency of the eight items of the C-OIDP was tested by performing inter-item correlation. A Bonferroni corrected alpha value was obtained by dividing α = 0.05 by the total number of comparison groups in this test. A test-retest reliability study of the C-OIDP was done by re-administration of the C-OIDP questionnaire to 10% of the sample (180 participants).

#### Assessment of C-OIDP

The Child-OIDP was evaluated by asking participants during the past three months how often the problems with your mouth and teeth caused you any difficulty with eating, speaking, cleaning your teeth, sleeping, smiling, your emotional state, schoolwork, and social contact. Each item was scored as 0 = never, 1 = once or twice per month, 2 = once or twice per week, and 3 = every day or nearly every day. The prevalence of oral impact for each item was computed by dichotomizing scores for each of the eight items into 0 = no impact (score 0) and 1 = with impact (scores 1–3). The overall impact (impact based on all eight items) was obtained by first computing the total of the eight dichotomized frequency scores (min 0, max 8) and then dichotomizing into 0 = no impact (score 0) and 1 = with impact (scores 1–8).

### Socio-demographic covariates

Participant’s sex was recorded as 1 = male and 2 = female, while age was calculated from the date of birth and dichotomized according to World Health Organization (WHO) adolescence categories into 10–14 (early adolescence) and 15–19 (mid and late adolescence)[14]. Parental education was recorded as 1 = no formal education, 2 = primary, 3 = secondary, and 4 = college or university and dichotomized into 1 = up to primary, 2 = secondary, and above. Socioeconomic status (SES) questions were adopted from a standardized International Wealth Index instrument (IWI) [15] and a recent demographic and health survey in Zambia [16]. The questions inquired about possession of one or more of the following assets: television, bicycle, car, plough, and phone. It also inquired on housing conditions such as the type of wall material used, such as mad or cement blocks, and the roof material used, such as animal dung, grass, iron sheets, or concrete. Other components were the type of toilet (flashing toilet, pit latrine, none), water source (piped water, borehole, shallow well), and access to electricity. The scoring of the items was done according to the weights of the items as prescribed by the index, which add up to 74.99553 and a constant of 25.00447. The total IWI scores were computed as a total of the IWI weight scores for the items (minimum 25.00447 and maximum 100), and thereafter the principal component analysis (PCA) was run. The first component, which accounts for the largest proportion of variance, was used as the wealth index. The 25th, 50th, and 75th percentiles of the first component scores guided the categorization of participants into quantiles as follows: below the 25th percentile as the 1st quantile, 25th to 50th percentile as the 2nd quantile, 50th to 75th percentile as the 3rd quantile, and above the 75th percentile as the 4th quantile. Dichotomization into two groups of SES was done by recategorizing the 1st to 3rd quantile as low to middle SES and the 4th quantile as high SES.

### Oral health-related behaviors

Oral health-related behaviors were assessed in terms of frequency of tooth brushing and use of fluoridated toothpaste per day and coded as {1 = I didn’t, 2 = I did but not every day, 3 = I did once a day, 4 = I did twice or more per more}, frequency of intake of added sugar-containing diet in the past 30 days coded as {1 = I didn’t take, 2 = occasionally per week, 3 = once per day, 4 = twice to four times per day, 5 = five times or more per day}. Tooth brushing and use of fluoridated toothpaste were combined to form one variable ‘tooth brushing using fluoridated toothpaste’ and recorded as {1 = less than 2 times per day and 2 = twice or more per day}. The frequency of intake of added sugar-containing food and drinks in the past 30 days was also combined to form a variable ‘frequency of intake of added sugary diet’ and recorded as {1 = 5 times or more per day and 2 = less than 5 times per day}. Dental visits in the previous year were coded as {1 = I didn’t attend, 2 = I attended once, 3 = I attended twice or more} and dichotomized as {1 = I didn’t attend and 2 = I attended once or more}.

### Self-reported oral health and oral symptoms

Self-reported oral health in terms of the health of gums and teeth was recorded as {1 = very poor, 2 = poor, 3 = good, 4 = very good} and dichotomized as {1 = poor, 2 = good}. Self-reported oral symptoms in the past three months were recorded as {1 = no event of painful tooth, 2 = at least one event of painful tooth}.

### Training and calibration of examiners on CAST instrument

The training of the principal investigator (SA), who is a specialist in restorative dentistry, was facilitated by an experienced epidemiologist (CKN). The training included instructions on how to conduct examinations, report findings, and interpret results, as well as the justification behind CAST codes and their descriptions. In the initial calibration stage, 20 clinical images representing two or more of each of the nine CAST codes were used. In the second calibration stage, 15 pre-selected adolescents with various CAST codes on one or more teeth were examined. The first five pre-selected adolescents were examined by each PI (SA) and the calibrator (CKN), and the findings were then compared. The differences were reviewed, and the second and third groups of five adolescents were then examined. Following the same procedure, the principal investigator (SA) trained and calibrated the examiners. A detailed explanation is provided in Appendix 3.

### Oral clinical examination

An oral examination was conducted inside the classrooms by four trained and calibrated dentists, with adolescents sitting on a desk opposite the classroom windows. Permanent teeth were examined according to the Caries Assessment Spectrum and Treatment (CAST) index [17] and coded as {0 = sound, 1 = sealant, 2 = restoration, 3 = caries in enamel, 4 = caries in dentine without distinct cavitation (discolored dentine visible through enamel), 5 = caries in dentine with distinct cavitation, 6 = caries in pulp, 7 = abscess or fistula, 8 = lost due to caries, 9 = others}. CAST 0–2 were categorized as healthy, CAST 3–4 as pre-morbidity, CAST 5 as morbidity, CAST 6–7 as severe morbidity, and CAST 8 as mortality. The prevalence of dental caries was defined according to the CAST manual as any participant with one or more teeth at CAST stages 3 to 7 [17].

### Statistical analysis

**Fig. 2:**
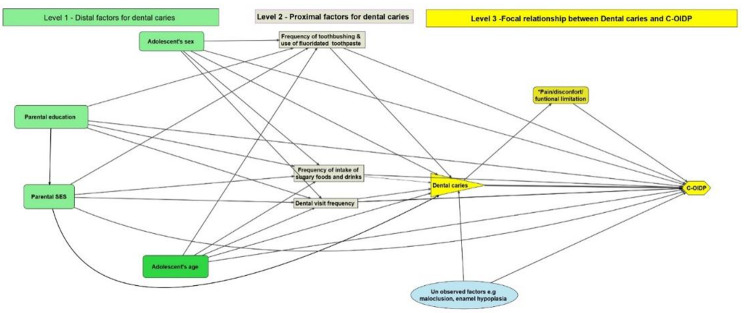
Directed acyclic graph (DAG) illustrating relationship between dental caries and C-OIDP

Data entry, cleaning, and analysis were done using Stata/SE (version 17). Descriptive data on independent variables, including self-reported and clinically determined dental caries, were dichotomized and summarized as frequencies and percentages. These variables included demographics of the adolescents, such as age and sex, parental/guardian socio-demographics, such as parental education and socioeconomic status, oral health-related behaviors, such as frequency of intake of added sugary foods, dental visits in the past year, and tooth brushing with fluoridated toothpaste. Inter-correlations between C-OIDP were reported as Spearman’s correlation coefficients. All the p values of the 28 pairwise correlations were compared with the Bonferroni corrected alpha value (p < 0.002). Cohen’s Kappa (n = 180) was used to evaluate the test-retest reliability of C-OIDP items by re-administering the inventory to every 10th participant at an interval of 10 days. Internal consistency reliability of C-OIDP was assessed by Cronbach’s alpha. The Kappa coefficient was used to assess the intra- and inter-examiner consistency in examining dental caries. The chi-square test was used to compare impacts among those with and without clinically assessed and self- reported dental caries. The minimal set of covariates for adjustment was assessed based on DAG [Fig. 2]. Dagitty software [18] was used to select the minimum set of covariates for adjustment of confounders. Hierarchical logistic regression (adjusted for clusters) was performed to examine the association between dental caries and C-OIDP. Distal factors (adolescents’ age and sex) were entered at the first stage. The proximal factors (tooth brushing using fluoridated toothpaste, frequency of consuming sugary diet and dental visits in the past year) were entered at the second stage, while dental caries was entered at the third stage. Adjusted odds ratios (AOR) with a 95% confidence interval (CI) were used to present the results.

### Directed Acyclic Graphs (DAGs)

The study used directed acyclic graphs (Fig. 2) to illustrate the underlying association between dental caries and C-OIDP while considering socio-demographic and oral health-related covariates that might influence the association [19]. In summary, dental caries was assumed to have a direct effect on C-OIDP. Proximal factors such as oral health-related behaviors in terms of tooth brushing and use of fluoridated toothpaste frequency, sugary food consumption, and dental visit frequency were assumed to have direct effects on C-OIDP and indirect effects through dental caries. Distal factors (socio-demographics) were assumed to have indirect effects on C-OIDP mediated through more proximal individual oral health-related behaviors. A detailed description of the assumed pathways in the DAG (Fig. 2) is provided in the appendices (Appendix 1).

## Results

### Sample characteristics

A total of 1,794 were enrolled in this study out of 1,909 eligible adolescents at the time of the study, making a participation rate of 94% (Fig. 1). The participants were aged between 11 and 19 years, with a mean age of 14.6 years SD (1.7). Table 1 presents the frequency distribution of participants’ socio-demographic and oral health-related behaviors, CAST clinical categories and prevalence, and their distribution according to C-OIDP status.

**Table 1:** Characteristics of study participant’s socio-demographics, oral health related behaviors, CAST clinical categories, CAST prevalence and their distribution according to C-OIDP status (n = 1794)

Socio-demographics		Frequency % (n)	C-OIDP		p value
			C-OIDP = 0	C-OIDP > 0	
Participants sex	male	46.0 (825)	70.7 (583)	29.3 (242)	0.076
	female	54.0 (969)	66.8 (647)	33.2 (322)	
Participants age	11–14	56.0 (1004)	71.6 (719)	28.4 (285)	0.002
	15–19	44.0 (790)	64.7 (511)	35.3 (279)	
Parental education	low (up to primary)	48.9 (876)	67.5 (591)	32.5 (285)	0.329
	Middle to high (secondary+)	51.1(918)	69.6 (639)	30.4 (279)	
Parental SES	low to middle	43.8 (785)	66.2 (520)	33.8 (265)	0.062
	High	56.2 (1009)	70.4 (710)	26.6 (299)	
**Oral health related behaviors**					
Sugary foods consumption per day	less than 5 times	81.8 (1468)	70.9 (1041)	29.1 (427)	< 0.001
	5 times or more	18.2 (326)	58.0 (189)	42.0 (137)	
Tooth brushing using fluoridated toothpaste per day	2 times or more	45.5 (816)	70.7 (577)	29.3 (239)	0.073
	less than 2 times	54.5 (978)	66.8 (653)	33.2 (325)	
Dental visit in the past 1 year	Attended	26.4 (474)	63.3 (300)	36.7 (171)	0.004
	did not attend	73.6 (1320)	70.5 (930)	29.5 (390)	
**Dental caries**					
CAST -clinical severity score	healthy (CAST 0–2)	51.1 (917)	80.0 (734)	20.0 (183)	< 0.001
	pre-morbidity (CAST 3)	24.6 (442)	69.0 (305)	31.0 (137)	
	morbidity (CAST 4–5)	15.2 (272)	50.7 (138)	49.3 (134)	
	severe morbidity (CAST 6–7)	6.4 (114)	31.6 (36)	68.4 (78)	
	mortality (CAST 8)	2.7 (49)	34.7 (17)	65.3 (32)	
CAST- caries prevalence	caries free (CAST 0–2 and 8)	53.8 (966)	77.7 (751)	22.3 (215)	< 0.001
	with caries (CAST3-7)	46.2 (828)	57.9 (479)	42.1 (349)	

Females constituted 54.0%, 56.0% were aged 11–14 years, and 56.2% were from high SES families. The distribution of participants’ oral health-related behaviors showed that 18.2% consumed sugary foods five times or more per day, 45.5% brushed their teeth using fluoridated toothpaste two times per day, and 26.4% visited a dentist in the previous year. A quarter (24.6%) had one or more teeth at the pre-morbidity stage, 15.2% at the morbidity stage, 6.4% at the severe morbidity stage, and 2.7% at the mortality stage. Dental caries prevalence, as determined by CAST, was 46.2%. Participants’ age (p = 0.002), frequency of eating sugary foods (p < 0.001), use of fluoridated toothpaste to brush their teeth (p < 0.001), dental visits in the past year (p < 0.001), clinical severity stage of CAST (p < 0.001), and prevalence of CAST (p < 0.001) showed differences in C-OIDP status. A painful tooth or teeth in the past three months was reported by 20.2% of the participants.

### Psychometric properties and prevalence of C-OIDP

Cronbach’s alpha score for all the items in the total sample was 0.8, while corrected item-total correlations ranged from 0.621 to 0.72. Inter-correlations between C-OIDP items in terms of Spearman’s correlation coefficients are presented in Table 2. Spearman’s correlation coefficients for the eight C-OIDP items ranged from 0.399 to 0.641. All the p values of the 28 pairwise correlations were less than the Bonferroni-corrected alpha value (p 0.002). The test-retest reliability Kappa coefficients of C-OIDP items (n = 180) ranged from 0.960 to 1.00. Internal consistency reliability in terms of Cohen’s Kappa of the C-OIDP inventory was 0.940. Table 3 shows the OIDP single item and overall C-OIDP by self-reported tooth health and clinically assessed dental caries. Poor self-reported dental health and dental caries were associated with reporting oral impacts across the eight C-OIDP components and the overall C-OIDP. The overall prevalence of oral impacts was 31.5%, and the highest impacts were with eating (26.5%), cleaning teeth (16.4%), and sleeping (12.5%). Social contact (8.1%) and emotional state (7.9%) were the least reported impacts.

**Table 2:** Spearman’s correlation of Child -OIDP performance items

Child OIDP item	Eating	Speaking	Cleaning teeth	Sleeping	Smiling	Emotional state	School	Social Contact
Eating	1							
Speaking	0.427	1						
Cleaning teeth	0.589	0.498	1					
Sleeping	0.543	0.512	0.552	1				
Smiling	0.441	0.480	0.488	0.478	1			
Emotional state	0.399	0.482	0.473	0.550	0.564	1		
School	0.482	0.546	0.515	0.593	0.503	0.595	1	
Social Contact	0.432	0.491	0.488	0.551	0.568	0.596	0.641	1

All correlations were less than the Bonferroni corrected α = 0.05/28 = 0.002

**Table 3:** Distribution of C-OIDP by single item and overall OIDP according to self-reported teeth health and clinically assessed dental caries

Variable	Individual Impacts > 0*								Overall* OIDP > 0
	Eating % (n)	Speaking % (n)	Cleaning teeth % (n)	Sleeping % (n)	% (n)	Emotion % (n)	School % (n)	Social Contact % (n)	
Self-reported teeth health									
Poor	61.6 (202)	26.9 (70)	39.6 (130)	31.7 (104)	26.5 (87)	21.0 (69)	22.6 (74)	22.6 (74)	69.5 (228)
Good	18.6 (273)	7.8 (119)	11.5 (168)	8.3 (122)	6.3 (92)	4.9 (72)	6.1 (89)	4.8 (71)	22.9 (336)
p value	< 0.001	< 0.001	< 0.001	< 0.001	< 0.001	< 0.001	< 0.001	< 0.001	< 0.001
Clinical assessed dental caries									
With caries	36.5 (302)	14.1 (117)	21.3 (176)	16.5 (137)	11.6 (96)	9.5 (79)	11.8 (98)	10.4 (86)	42.1 (349)
No caries	17.9 (173)	7.5 (72)	12.6 (122)	9.2 (89)	8.6 (83)	6.4 (62)	6.7 (65)	6.1 (59)	22.3 (215)
p value	< 0.001	<0.001	< 0.001	<0.001	0.034	0.014	<0.001	< 0.001	< 0.001

Note *Only percentage of participants with impacts (C-OIDP > 0) is provided. The percentage of participants without impact = 100%-value provided

### Minimal set of covariates for adjustment based on the dagitty software

It was assumed that proximal factors (regular use of fluoridated toothpaste, frequent consumption of sugary foods, and previous year’s dental visits) and distal factors (parental SES and education, adolescents’ age and sex) would influence the association between dental caries and C-OIDP. After entering the variables into the DAGitty v3.0 software, the minimum necessary adjustment sets for estimating the overall impact of dental caries on C-OIDP were the following: adolescent age, adolescent sex, frequency of tooth brushing with fluoridated toothpaste, frequency of consumption of sugary foods per day, and dental visit within the previous year.

### Hierarchical logistic regression analyses adjusted for clusters (schools)

Table 4 shows the hierarchical logistic regression model with the adjusted odds ratio (AOR) and 95% confidence interval (95% CI) of the predictors of C-OIDP. Age was associated with C-OIDP, such that older adolescents aged 15–19 years were 1.4 times (AOR 1.4; 95% CI 1.1–1.8) more likely to report impacts than those in the age group 10–14 at stage 1. Entering oral health-related behaviors in stage 2 revealed an association between C-OIDP and age (AOR 1.4, 95% CI 1.1–1.9), sugary food frequency per day (AOR 1.8, 95% CI 1.3–2.5), and dental visit per year (AOR 0.7, 95% CI 0.5–0.8). Age and sugary food frequency maintained an association with C-OIDP after entering dental caries in stage 3. The odds of reporting oral impacts were 1.4 times (AOR 1.4, 95% CI 1.1, 1.9) and 1.7 times (AOR 1.7, 95% CI 1.2, 2.4) higher among participants aged 15–19 years and those consuming sugary foods five times or more per day, respectively, than their counterparts. Participants who visited a dentist at least once per year were 40% (AOR 0.6, 95% CI 0.5–0.7) less likely to report C-OIDP. The participants with dental caries were 2.6 times (AOR 2.6, 95% CI 2.1, 3.4) more likely to report oral impacts than those without caries.

**Table 4:** Hierarchical Logistic regression model of predictors of the association between dental caries and Child -OIDP (Cluster adjusted)

Covariates		Stage 1 R^2^ = 0.006		Stage 2 R^2^ = 0.099		Stage 3 R^2^ = 0.060	
		OR (95% CI)	p value	OR (95% CI)	p value	OR (95% CI)	p value
Adolescents’ Sex	Male	1		1		1	
	Female	1.2 (0.9, 1.6)	0.203	1.2 (0.9, 1.6)	0.158	1.1 (0.8, 1.5)	0.400
Adolescents’ Age	10–14	1		1		1	
	15–19	1.4 (1.1, 1.8)	0.026	1.4 (1.1, 1.9)	0.010	1.4 (1.1, 1.9)	0.029
Toothbrushing with fluoride toothpaste	2 times or more per day			1		1	
	Less than 2 times per day			1.3 (1.1, 1.7)	0.016	1.4 (1.1, 1.8)	0.007
Sugary foods consumption per day	Less than five time per day			1		1	
	Five times or more per day			1.8(1.3, 2.5)	0.001	1.7 (1.2, 2.4)	< 0.001
Dental visit per year	Did not attend			1		1	
	Attended at least once			0.7 (0.5, 0.8)	< 0.001	0.6 (0.5, 0.7)	< 0.001
Dental caries	No caries					1	
	With Caries					2.6 (2.1, 3.4)	< 0.001

## Discussion

The study revealed acceptable psychometric properties of the C-OIDP index and a positive association between dental caries and the C-OIDP. The severe stages of the caries assessment and treatment spectrum (CAST) were more likely to be associated with C-OIDP. The prevalence of C-OIDP was high, and the most frequent impacts were more related to difficulties with eating and cleaning teeth. Participants with dental caries were more likely to experience C-OIDP than those without dental caries after adjustment for confounding variables identified by the use of DAGs. Painful teeth in the past three months were highly associated with increased oral impacts.

Interpretation of the findings needs to be done with consideration of the following limitations: This was a cross-sectional study that ascertains exposure and outcome at the same point in time, so causal inference cannot be established. The results on self-reported oral health-related behaviors are subject to social desirability bias, similar to any other behavioral-related study. [20]. An attempt to reduce this bias was done through the use of a self-administered anonymous questionnaire filled in by participants under the supervision of research assistants in a large classroom where they could not easily see each other’s responses. Participants could have responded or chosen socially desirable responses and therefore they might have under-reported poor oral health-related behaviors such as frequent consumption of sugary foods or over-reporting good oral health behaviors such as visiting a dentist for checkup or tooth brushing using fluoridated toothpaste. Information on oral health-related behaviors is also subject to recall bias, but efforts were done to minimize the bias by structuring the questions and options in a way that makes them easy to recall, as well as by limiting the recall period for eating sugary foods and using fluoridated toothpaste to one month. One year was chosen as the recall interval for the last dental visit since it is thought that bias is unlikely to be introduced within that time frame. The accuracy of the Caries Assessment and Treatment Spectrum in the detection of none cavitated early carious lesions is limited equally between those with and without C-OIDP due to the inability to use compressed air to dry a tooth surface in the field environment. Thus, the possibilities of non-differential misclassification cannot be ignored. The findings of this study may not be able to be generalized to Zambian adolescents due to the non-random selection of Copperbelt province. However, the findings may give a picture of other cosmopolitan provinces within Zambia. A large sample size drawn from randomly selected districts and schools of Copperbelt province contributes to the strength of our findings. Training and calibration of examiners on the Caries Assessment and Treatment (CAST) tool before the collection of exposure data reduced variability and the chances of misclassifying exposure [17]. High test-retest reliability and internal consistency of the C-OIDP also contribute to the strength of our findings and confirm the applicability of the tool among Zambian adolescents.

In the current study, the test-retest results and internal consistency were both within acceptable standards. The internal consistency exceeded the threshold (0.70), which is regarded as a reliable indicator of an instrument’s performance [21]. The findings demonstrate that each participant’s replies to the eight C-OIDP items were consistent, and as a result, they are probably capable of appropriately assessing C-OIDP [21]. Internal consistency values for the C-OIDP index ranging from 0.5 to 0.9 have been reported in previous studies [22–24]. Test-retest reliability for the C-OIDP items, which assesses an instrument’s consistency over time, ranged between good and satisfactory, indicating that the results are repeatable over time [21].

The findings of this study show a high prevalence of dental caries and C-OIDP as well as a significant association between the two. The high frequency of dental caries, particularly severe stages, may have contributed to the high prevalence of C-OIDP. The high prevalence of C-OIDP is consistent with some studies in Sub-Saharan Africa (SSA) [23, 24]. However, considerably lower [8] and higher [4, 25, 26] prevalence of C-OIDP than those in the current study were reported among adolescents in other studies. High prevalence of C-OIDP up to 100% are reported in some high-income countries [2]. Similar to other previous studies C-OIDP items related to pain and functional limitation such as difficulty with eating, cleaning teeth, and sleeping which are more likely to be reported by individuals with dental caries at advanced stages were the most frequently reported individual items of C-OIDP [4]. The higher frequencies of these items could be attributed to the fact that these daily activities are more likely to be affected in a person with a painful tooth [27]. The order in the rank of the most reported C-OIDP items is in line with that of other previous studies in Sub–Saharan Africa [4].

The results of the current study call for all stakeholders involved in oral health, including the ministries of health, education, and youth and non-governmental organizations, to take deliberate action to provide preventive oral health measures to this important group, which comprises about 23% of the Zambian population [16]. The high prevalence of dental caries and C-OIDP are likely to persist during their adulthood if preventive measures are not taken [28]. The measures may include addressing the problem by focusing on reducing the frequency of intake of sugary-containing foods and monitoring early cavitated teeth as primary preventive measures. Addressing dental caries in school environments such as the provision of simple restorative procedures (Atraumatic Restorative Treatment) and emergency treatment may be considered rather than waiting for adolescents to visit dental clinics.

## Conclusion

Dental caries was associated with high reporting of C-OIDP, and C-OIDP prevalence was high among participants in the severe stages of the caries process. The English version of the C-OIDP demonstrated adequate psychometric characteristics for assessing OHRQoL among Zambian adolescents.

## Electronic supplementary material

Below is the link to the electronic supplementary material.

Supplementary Material 1

Supplementary Material 2

Supplementary Material 3

Supplementary Material 4

## Data Availability

The anonymous data will be available from the authors upon request and with permission of the Zambia Health Research Authority and Muhimbili University of Health and Allied Sciences, Tanzania.

## Abbreviations

CAST: Caries Assessment and Treatment Spectrum
COHIP SF19: Child Oral Health Impact Profile Short Form 19
C-OIDP: Child -Oral Impact on Daily Performance
DAG: Directed Acyclic Graph
DEBS: District Education Board Secretary
DHS: Demographic Health Survey
IRB: Institutional Review Board
IWI: International Wealth Index
MUHAS: Muhimbili University of Health and Allied Sciences
NHRA: National Health Research Authority
OHRQoL: Oral Health Related Quality of Life
OIDP: Oral Impact on Daily Performance
RCFT: Randomized controlled field trial
SES: Socio-economic status
SSA: Sub Saharan Africa
TDRC: Tropical Disease Research Centre
WHO: World Health Organization
