# Supplementary material for: Assessing association of dental caries with child oral impact on daily performance; a cross-sectional study of adolescents in Copperbelt province, Zambia
Source: Health Qual Life Outcomes. 2023 May 18;21:47. doi: 10.1186/s12955-023-02127-9 (PMC10193792; doi:10.1186/s12955-023-02127-9)
Supplement: Supplementary file 1 — Supplementary Material 1 [file 12955_2023_2127_MOESM1_ESM.docx]

| **S/No** | **School name** | **Number of participants** | **% of total** |
| --- | --- | --- | --- |
|  | Chifubu A and B | 51 | 2.8 |
|  | Targagan High School | 76 | 4.2 |
|  | Mpongwe day | 65 | 3.6 |
|  | Kanyenda Sec | 133 | 7.4 |
|  | Temweni | 101 | 5.6 |
|  | Malasha | 64 | 3.6 |
|  | Kanshenshi sec | 44 | 2.5 |
|  | Milemu | 48 | 2.7 |
|  | Masala | 43 | 2.4 |
|  | Yengwe | 95 | 5.3 |
|  | Butikiri Combined | 81 | 4.5 |
|  | Chipulukusu | 47 | 2.6 |
|  | Kanini | 52 | 2.9 |
|  | Kanshenshi Combined | 61 | 3.4 |
|  | Ibenga SDA | 91 | 5.1 |
|  | Kawama | 58 | 3.2 |
|  | Ndola stem school | 86 | 4.8 |
|  | Mupapa sec | 113 | 6.3 |
|  | Fiwale sec | 101 | 5.6 |
|  | Chiwala stem school | 120 | 6.7 |
|  | Kafulafuta sec | 144 | 8.0 |
|  | Masaiti sec | 120 | 6.7 |
|  | **Total** | **1794** | **100.0** |
