# Supplementary material for: Assessing association of dental caries with child oral impact on daily performance; a cross-sectional study of adolescents in Copperbelt province, Zambia
Source: Health Qual Life Outcomes. 2023 May 18;21:47. doi: 10.1186/s12955-023-02127-9 (PMC10193792; doi:10.1186/s12955-023-02127-9)
Supplement: Supplementary file 2 — Supplementary Material 2 [file 12955_2023_2127_MOESM2_ESM.docx]

**Training and Calibration of examiners**

**Training and calibration of the principal investigator**

Training of the principal investigator (SA) who is a specialist in restorative dentistry was facilitated by an experienced epidemiologists (CKN) through interactive power point presentation, discussion and clinical examination of adolescents. In respect to the CAST index the training was done following CAST manual guideline. The training included rationale of CAST codes and their description, rationale of the index, ergonomic guidelines and instructions on how to perform examination, reporting and interpreting the findings. Twenty clinical photographs representing two or more of each code of the nine CAST codes were used (Figures) in the first stage of calibration. The PI and the calibrator (CKN) agreed on 18 out of 20 coded (90%).

The second stage of calibration involved examination of fifteen pre -selected 10-14 years old adolescents with different CAST codes on one or more teeth at Matosa secondary school, in Ubungo district, Dar es Salaam Tanzania. Examination was conducted in a classroom with the aid of artificial light. The adolescents were instructed to brush their teeth before examination. Ergonomic principles were observed whereby the adolescents were examined while lying almost flat on a bench with their heads on a pillow placed on examiners thighs. The examiners sat behind the patient head at 10-12 O’clock position. The recorder sat on the other side facing the subject’s oral cavity. Removal of remaining plaque and drying if teeth was done using gauze. and The Examination of the first 5 pre-selected adolescents was done by each PI (SA) and the calibrator (CKN) then results compared. An agreement of 87.9 % was found with the most disagreement in scores 3, 4 and 5. The differences were discussed and then examination of the second batch of 5 adolescents was done which yielded an 91.4 % agreement. Final agreement of 97.2% was achieved in the last group of 5 adolescents.

**Training and calibration of the examiners**

Training of the examiners was done by the principal investigator (SA) through interactive power point presentation, discussion and clinical examination of adolescents. A total of twelve potential examiners and data recording assistants were trained at Copperbelt University, Ndola Zambia. Four examiners and four assistants were finally selected based on the final score.

Results of clinical photograph calibration and clinical examination vs principal investigator (SA).

| ID | Clinical Photographs Scores | | | Average Clinical Score |
| --- | --- | --- | --- | --- |
|  | 1st Score | % agreement | 2nd score |  |
| 1 | 13/23 | 56% | 100% | 90% |
| 2 | 15/23 | 65% | 95.60% | 89% |
| 3 | 14/23 | 60.80% | 100% | 88% |
| 4 | 15/23 | 65.20% | 84.80% | 79% |
| 5 | 15/23 | 65.20% | 84.80% | 76% |
| 6 | 14/23 | 60% | 91% | 74% |
| 7 | 14/23 | 60% | 100% | 89% |
| 8 | 12/23 | 52% | 96% | 88% |
| 9 | 12/23 | 52% | 76% | 72% |
| 10 | 14/23 | 60% | 74% | 80% |
| 11 | 14/23 | 60% | 96% | 87% |
| 12 | 11/23 | 56% | 96% | 90% |

The most areas of disagreement were on CAST 3 and 4 and high agreement were on CAST 5,6, 7 and 8.
