# Supplementary material for: Assessing association of dental caries with child oral impact on daily performance; a cross-sectional study of adolescents in Copperbelt province, Zambia
Source: Health Qual Life Outcomes. 2023 May 18;21:47. doi: 10.1186/s12955-023-02127-9 (PMC10193792; doi:10.1186/s12955-023-02127-9)
Supplement: Supplementary file 3 — Supplementary Material 3 [file 12955_2023_2127_MOESM3_ESM.docx]

**Appendix 1 - Description of the direct paths in the DAG and references**

Figure 2 shows a Directed Acyclic Graph (DAG) illustrating the relationship between dental caries as the main exposure and Child Oral Impact on Daily Performance (C-OIDP) as outcome based on the conceptual framework of dental caries (1) and conceptual model of patient outcomes (2). The DAG was constructed to visualize the association between dental caries and C-OIDP. Initially, all possible direct paths between variables included were evaluated for plausibility based on theory and empirical evidence. Education is one of the main factors that determine the job position and income of an individual which reflects socioeconomic status (SES) (3). Several studies describe the influences of socioeconomic status on oral health-related behaviors such as tooth brushing using fluoridated toothpaste and frequency of intake of a sugary diet (4–8). Sex and age also affect oral health behaviors (7,9–13). Dental caries is a behavioral-related oral disease and therefore the behaviors are major predictors of dental caries (12,14). Parental socioeconomic status has been shown to influence child OIDP where high income is associated with better OHRQoL (15). Evidence of the relationship between gingivitis and oral impact on daily performance is provided in a cohort study that found gingivitis was associated with higher oral impacts (16). Parental SES influences oral health-related behaviors such as toothbrushing (17) and use of fluoridated toothpaste and frequency of intake of sugary diet which in turn affect the occurrence of dental caries (18). In low to middle-income countries where the main reason for dental visits is for relief of pain and not preventive, a dental visit is likely to reduce number of carious teeth but increase number of extracted teeth due to caries (19). Evidence of the relationship between sugary diet and C-OIDP is provided by a study in Turkey that reports high impacts among those taking a sugary diet more frequently (20). Based on the above scientific evidence we evaluated the direct and indirect association between dental caries and C-OIDP using a DAG (Figure 1). The indirect pathway from dental caries to C-OIDP is through causing pain and subsequently influencing OHRQoL. Dental caries is the most common cause of pain and discomfort (21). A study which evaluated oral impact on daily performance and recent use of dental services in school children revealed increased use of dental services among those with oral impacts (22). The pathways for the assumed saturated DAG are illustrated below.

**Evaluated pathways**

**Open front door paths (causal pathway):**

1. Dental caries → C-OIDP
2. Dental caries → Pain/discomfort/Impaired esthetics arising from caries → C-OIDP

All the two front door paths are open, the first is a direct pathway while the second is an indirect pathway through pain/discomfort/Impaired esthetics as mediators. No need for adjustment of pain discomfort/functional impairment, otherwise it will reduce the detrimental effect of dental caries on C-OIDP.

B**ack door paths**

1. Dental caries ← toothbrushing & toothpaste use → C-OIDP

(Open backdoor path: adjust for toothbrushing & toothpaste use)

1. Dental caries ← toothbrushing & toothpaste use ← Adolescent’s sex→ C-OIDP

(Open backdoor path: adjust for toothbrushing & toothpaste use and Adolescents sex)

1. Dental caries ← toothbrushing & toothpaste use ← Adolescent’s age→ C-OIDP

(Open backdoor path: adjust for toothbrushing & toothpaste use and Adolescents age)

1. Dental caries ← toothbrushing & toothpaste use ← Parental education→ C-OIDP

(Open backdoor path: adjust for toothbrushing & toothpaste use and Parental education)

1. Dental caries ← toothbrushing & toothpaste use ← Parental SES→ C-OIDP

(Open backdoor path: adjust for toothbrushing & toothpaste use and Parental SES)

1. Dental caries ← toothbrushing & toothpaste use ← Adolescent’s sex→ Sugary diet intake→ C-OIDP

(Open backdoor path: adjust for toothbrushing & toothpaste use, Adolescent’s sex and Sugary diet intake)

1. Dental caries ← toothbrushing & toothpaste use ← Adolescent’s sex→ Dental visit→ C-OIDP

(Open backdoor path: Adjust for toothbrushing & toothpaste use, Adolescent’s sex and Dental visit)

1. Dental caries ← toothbrushing & toothpaste use ← Adolescent’s age→ Sugary diet→ C-OIDP

(Open backdoor path: adjust for toothbrushing & toothpaste use, Adolescent’s age and Sugary diet intake)

1. Dental caries ← toothbrushing & toothpaste use ← Adolescent’s age→ Dental visit→ C-OIDP

(Open backdoor path: Adolescents age, toothbrushing & toothpaste use and dental visit)

1. Dental caries ← toothbrushing & toothpaste use ← Parental education→ Parental SES→ C-OIDP

(Open backdoor path: adjust for toothbrushing & toothpaste use, Parental education and Parental SES)

1. Dental caries ← toothbrushing & toothpaste use ← Parental education→ Parental SES→ Sugary diet intake ← Adolescents age→ C-OIDP

(Open backdoor path: adjust for toothbrushing & toothpaste use, Parental education, Parental SES, Sugary diet intake and Adolescents age,)

1. Dental caries ←Dental visit ←Adolescent’s age → C-OIDP

(Open backdoor path: adjust for Adolescents age and Dental visit)

1. Dental caries ←Dental visit ←Parental SES ← Parental education→ use of toothbrushing & toothpaste → C-OIDP

(Open backdoor path: Adjust for dental visit, Parental SES, Parental education and use of toothbrushing & toothpaste)

1. Dental caries ←Sugary diet intake→ C-OIDP

(Open backdoor path: adjust for Sugary diet intake)

1. Dental caries ←Sugary diet intake→ Adolescent’s sex →C-OIDP

(Open backdoor path: adjust for Sugary diet intake and Adolescent’s sex)

1. Dental caries ←Sugary diet intake ← Parental education →C-OIDP

(Open backdoor path: adjust for Sugary diet intake and Parental education)

1. Dental caries ←Sugary diet intake ← Parental SES← Parental education →C-OIDP

(Open backdoor path: adjust for Sugary diet intake, Parental SES and Parental education)

1. Dental caries ←Sugary diet intake ← Parental SES← Parental education →C-OIDP

(Open backdoor path: adjust for Sugary diet intake, Parental SES and Parental education)

1. Dental caries ←Sugary diet intake ← Sex →Toothbrushing & use of toothpaste →C-OIDP

(Open backdoor path: adjust for Sugary diet intake, Sex, Toothbrushing & use of toothpaste)

Minimum adjustment set for DAG after entry into DAGGity sofware: Adolescent’s sex, Adolescent’s age, Toothbrushing & use of toothpaste, Sugary diet intake, and dental visit

**References**

1. Petersen, P. E. (2005). Sociobehavioural risk factors in dental caries–international perspectives. *Community dentistry and oral epidemiology*, *33*(4), 274-279.

2. Wilson, I. B., & Cleary, P. D. (1995). Linking clinical variables with health-related quality of life: a conceptual model of patient outcomes. *Jama*, *273*(1), 59-65.

3. Stryzhak, O. (2020). The relationship between education, income, economic freedom and happiness. In *SHS Web of Conferences* (Vol. 75, p. 03004). EDP Sciences.

4. Broadbent, J. M., Zeng, J., Foster Page, L. A., Baker, S. R., Ramrakha, S., & Thomson, W. M. (2016). Oral health–related beliefs, behaviors, and outcomes through the life course. *Journal of dental research*, *95*(7), 808-813.

5. Buldur, B., & Güvendi, O. N. (2020). Conceptual modelling of the factors affecting oral health‐related quality of life in children: A path analysis. *International Journal of Paediatric Dentistry*, *30*(2), 181-192.

6. Gomes, A. C., Rebelo, M. A. B., de Queiroz, A. C., de Queiroz Herkrath, A. P. C., Herkrath, F. J., Rebelo Vieira, J. M., ... & Vettore, M. V. (2020). Socioeconomic status, social support, oral health beliefs, psychosocial factors, health behaviours and health-related quality of life in adolescents. *Quality of life research*, *29*(1), 141-151.

7. Elamin, A., Garemo, M., & Mulder, A. (2021). Determinants of dental caries in children in the Middle East and North Africa region: a systematic review based on literature published from 2000 to 2019. *BMC Oral Health*, *21*(1), 1-30.

8. Bombert, F., Manso, A. C., Ferreira, C. S., Nogueira, P., & Nunes, C. (2018). Sociodemographic factors associated with oral health in 12-year-old adolescents: hygiene behaviours and health appointments. A cross-sectional national study in Portugal. *International dental journal*, *68*(5), 327-335.9. Lukacs JR. Sex differences in dental caries experience: Clinical evidence, complex etiology. Clin Oral Investig. 2011;15(5):649–56.

10. López, R., Smith, P. C., Göstemeyer, G., & Schwendicke, F. (2017). Ageing, dental caries and periodontal diseases. *Journal of clinical periodontology*, *44*, S145-S152.

11. Kramer, A. C. A., Pivodic, A., Hakeberg, M., & Östberg, A. L. (2019). Multilevel analysis of dental caries in Swedish children and adolescents in relation to socioeconomic status. *Caries research*, *53*, 96-106.

12. Ndagire, B., Kutesa, A., Ssenyonga, R., Kiiza, H. M., Nakanjako, D., & Rwenyonyi, C. M. (2020). Prevalence, severity and factors associated with dental caries among school adolescents in Uganda: a cross-sectional study. *Brazilian dental journal*, *31*, 171-178.13. Alvarez-Azaustre, M.P.; Greco, R.; Llena, C. Oral Health-Related Quality of Life in Adolescents as Measured with the Child-OIDP Questionnaire: A Systematic Review. *Int. J. Environ. Res. Public Health* 2021; *18*:12995

14. Jepsen S, Blanco J, Buchalla W, Carvalho JC, Dietrich T, Dörfer C, et al. Prevention and control of dental caries and periodontal diseases at individual and population level: consensus report of group 3 of joint EFP/ORCA workshop on the boundaries between caries and periodontal diseases. J Clin Periodontol. 2017;44: S85–93.

15. Kumar S, Kroon J, Lalloo R. A systematic review of the impact of parental socio-economic status and home environment characteristics on children's oral health related quality of life. Health Qual Life Outcomes. 2014; 12:41.

16. Ortiz FR, Sfreddo CS, Coradini AGM, Fagundes MLB, Ardenghi TM. Gingivitis influences oral health-related quality of life in adolescents: Findings from a cohort study. Revista Brasileira de Epidemiologia. 2020; 23:1–12.

17. Acuña-González GR, Casanova-Sarmiento JA, Islas-Granillo H, Márquez-Rodríguez S, Benítez-Valladares D, Mendoza-Rodríguez M, et al. Socioeconomic Inequalities and Toothbrushing Frequency among Schoolchildren Aged 6 to 12 Years in a Multi-Site Study of Mexican Cities: A Cross-Sectional Study. Children. 2022;9(7):1069.

18. Moynihan P. Sugars and dental caries: Evidence for setting a recommended threshold for intake. Advances in Nutrition. American Society for Nutrition; 2016; 7:149–56.

19. Mbawalla HS, Masalu JR, Åstrøm AN. Socio-demographic and behavioural correlates of oral hygiene status and oral health related quality of life, the Limpopo - Arusha school health project (LASH): A cross-sectional study. BMC Pediatr. 2010;10(1):87.

20. Peker K, Eden E, Ak AT, Uysal Ö, Bermek G. Psychometric evaluation of the child oral impacts on daily performances (C-OIDP) for use in Turkish primary school children: A cross sectional validation study. BMC Oral Health. 2020 ;20(1).

21. Boeira GF, Correa MB, Peres KG, Peres MA, Santos IS, Matijasevich A, et al. Caries is the main cause for dental pain in childhood: Findings from a birth cohort. Caries Res. 2012;46(5):488–95.

22. Monsantofils, Monica and Eduardo Bernabé. “Oral impacts on daily performances and recent use of dental services in schoolchildren.” International journal of paediatric dentistry 24 6 (2014): 417-23 .
